# AB_5_ Enterotoxin-Mediated Pathogenesis: Perspectives Gleaned from Shiga Toxins

**DOI:** 10.3390/toxins14010062

**Published:** 2022-01-16

**Authors:** Erika N. Biernbaum, Indira T. Kudva

**Affiliations:** 1 Oak Ridge Institute for Science and Education, Oak Ridge, TN 37830, USA; Erika.Biernbaum@usda.gov; 2 Food Safety Enteric Pathogens Research Unit, National Animal Disease Center, United States Department of Agriculture (USDA), Ames, IA 50010, USA

**Keywords:** AB-type toxins, STEC, Stx, pathogenesis

## Abstract

Foodborne diseases affect an estimated 600 million people worldwide annually, with the majority of these illnesses caused by Norovirus, *Vibrio*, *Listeria*, *Campylobacter*, *Salmonella*, and *Escherichia coli*. To elicit infections in humans, bacterial pathogens express a combination of virulence factors and toxins. AB_5_ toxins are an example of such toxins that can cause various clinical manifestations, including dehydration, diarrhea, kidney damage, hemorrhagic colitis, and hemolytic uremic syndrome (HUS). Treatment of most bacterial foodborne illnesses consists of fluid replacement and antibiotics. However, antibiotics are not recommended for infections caused by Shiga toxin-producing *E. coli* (STEC) because of the increased risk of HUS development, although there are conflicting views and results in this regard. Lack of effective treatment strategies for STEC infections pose a public health threat during outbreaks; therefore, the debate on antibiotic use for STEC infections could be further explored, along with investigations into antibiotic alternatives. The overall goal of this review is to provide a succinct summary on the mechanisms of action and the pathogenesis of AB_5_ and related toxins, as expressed by bacterial foodborne pathogens, with a primary focus on Shiga toxins (Stx). The role of Stx in human STEC disease, detection methodologies, and available treatment options are also briefly discussed.

## 1. Introduction

Foodborne illnesses are a common occurrence worldwide. The Centers for Disease Control and Prevention (CDC) estimates that 1 in 6 Americans, or 48 million people, acquire a foodborne illness each year in the United States [1], while the World Health Organization (WHO) reports that 1 in 10 people, or 600 million, obtain a foodborne illness annually worldwide [2]. Foodborne illnesses contribute to more than 420,000 deaths globally each year and children under the age of 5 years are often prone to obtaining such diseases, representing 40% of the reported cases [2]. Annual treatment for foodborne illnesses and their related diseases costs an estimated USD 15 billion, as per the World Bank, with productivity losses estimated around USD 95.2 billion [3].

Transmission of foodborne pathogens occurs via the fecal–oral route or by ingesting contaminated drinks, fresh produce, unpasteurized milk, or undercooked meat and poultry. The CDC lists contaminated produce, such as leafy vegetables, eggs, raw milk, poultry, beef, and pork, as the most common sources to harbor such pathogens [4]. Symptoms during illness progression can range anywhere from fever, nausea, abdominal pain, diarrhea, and vomiting to kidney failure and death in extreme cases. Commonly implicated organisms include Norovirus, *Vibrio*, *Listeria*, *Campylobacter*, *Salmonella*, *Escherichia coli*, *Shigella*, and *Yersinia*, while other pathogens cause sporadic infections [1]. Of these, the Gram-negative bacteria naturally inhabit the intestinal tracts of animals, leading to their wide distribution in soil, water, and sewage. These bacterial pathogens can cause urinary tract infections (UTIs), bacteremia, pneumonia, and meningitis, in addition to foodborne illnesses [5,6].

The CDC estimates of current annual incidences of reported foodborne infections in the United States as a result of common Gram-negative bacterial pathogens are as follows: *Campylobacter* accounts for 1.5 million cases, non-typhoidal *Salmonella* for 1.35 million, Shiga toxin-producing *E. coli* (STEC) for more than 265,000, *Shigella* for 450,000, and *Yersinia* for 117,000 [7]. Globally, the median incidence in 2010 for *Campylobacter* gastroenteritis was 95.6 million, *E. coli* infections were around 111.5 million, and *Shigella* caused 51 million cases worldwide [8]. Non-typhoidal *Salmonella* global incidence was reported to be around 93.8 million in 2010 [9] and *Yersinia* resulted in 6839 cases in the European Union in 2014 [10]. Treatment of these bacterial infections has proven to be difficult over time, with increasing resistance against beta-lactams and carbapenem antibiotics, in part through horizontal gene transfer, possibly leading to a longer infection duration [11,12].

To cause such infections, bacterial pathogens express several virulence factors to aid in colonization and invasion, that in turn, trigger a cascade of specific reactions inside host cells that ultimately lead to host illness. The virulence factors expressed are responsible for adhesion, colonization, invasion, protection, immune evasion, and nutrient competition. For instance, the *spv* operon of the non-typhoid *Salmonella* species contains the genes *spvR, spvB,* and *spvC* that are responsible for intracellular survival, host macrophage apoptosis, repression of the host intestinal inflammatory response, prolonging infection by activating host actin degradation and preventing formation of F-actin, altering actin cytoskeleton, and irreversibly inhibiting MAP kinases [13]. *Shigella* species encode a serine protease, *sepA,* that disrupts the host intestinal epithelial barrier, allowing invasion of the basolateral pole, leading to fluid accumulation, a strong inflammatory response, and infection [14]. *E. coli*, such as enteropathogenic *E. coli* (EPEC) and enterohemorrhagic *E. coli* (EHEC), express a variety of virulence factors, such as those found on the pathogenicity island locus of enterocyte effacement (LEE), that encode a type-III secretion system, an adhesin (intimin), and a translocated intimin receptor (Tir) for colonization and adherence to host intestinal epithelial cells by forming attachments and effacing lesions [15]. In addition, enteroaggregative (EAEC) and atypical enteropathogenic *E. coli* secrete a serine protease plasmid-encoded toxin (Pet) responsible for spectrin and alpha-fodrin cleavage, resulting in host cell cytoskeletal retraction, mucosal inflammation, and secretory diarrhea [16,17]. While a combination of virulence factors is important for the survival of pathogenic bacteria inside a host, toxins play a major role in disease pathogenesis, particularly AB-type toxins, for which other types of virulence factors set the stage. Toxins damage host cells and tissues, invoke an immune response while evading detection, and cause life-threatening illnesses. Hence, the goal of this review is to succinctly summarize AB toxin-mediated pathogenesis, with a focus on Shiga toxins—the characteristic AB_5_ toxin produced by STEC.

## 2. AB-Type Toxins

Several bacteria possess AB-type toxins (Table 1) that elicit various diseases in humans, like diarrhea and other gastrointestinal issues, and play a crucial role in disease development. Although AB-type toxins are important for causing disease symptoms, their evolution has not yet been fully deduced. A few studies have shown, however, that phages contribute to the emergence of toxigenic bacteria by passing on their genetic material, such as toxin genes. For instance, the genes encoding cholera toxin, an AB_5_ toxin secreted by *Vibrio cholerae*, have been found on filamentous bacteriophage CTXφ [18], and ArtAB, secreted by *S. enterica*, can be found on various prophages depending upon the serovar [19,20]. AB-type toxins may have also arisen from recombination during horizontal gene transfer or gene-shuffling events, such as insertions, deletions, or point mutations [21]. While the evolution of AB-type toxins remains elusive, their structure is well-known and is key to understanding their role in bacterial pathogenesis. 

### 2.1. AB Toxins

**Figure 1:**
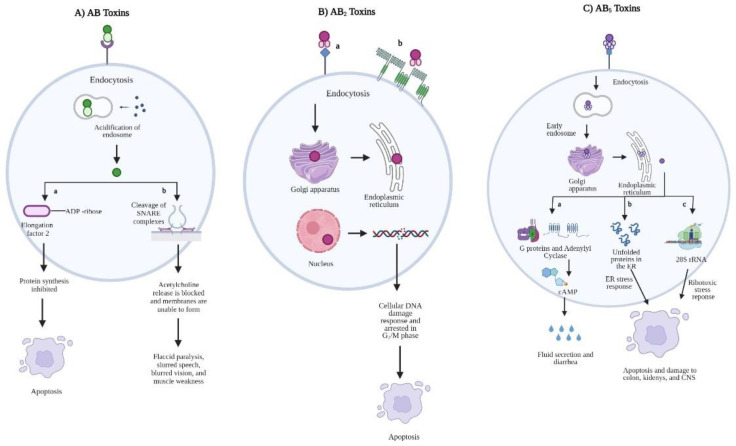
Comparative mode of action of AB-type toxins. (**A**) AB toxins. The B subunit of AB toxins binds to targeted host cell receptors to initiate receptor-mediated endocytosis. The endosome then becomes acidified and the A and B subunits disassociate. In the case of the diphtheria toxin and exotoxin A of *Pseudomonas aeruginosa*, the A subunit targets elongation factor 2 to inhibit host cell protein synthesis and cause apoptosis (a). The A subunit (light chain) of botulinum neurotoxins cleaves specific proteins in soluble *N*-ethylmaleimide–sensitive factor-activating protein receptor (SNARE) complexes, blocking the release of acetylcholine from presynaptic nerve endings (b). This also inhibits neurotransmitter-containing vesicles from fusing to presynaptic membranes, resulting in flaccid paralysis, muscle weakness, blurred vision, and slurred speech; (**B**) AB_2_ toxins. The B subunit of AB_2_ toxins binds to specific host cell ganglioside receptors (a) or to membrane lipid raft microdomains (b) to allow the A subunit to become endocytosed. The A subunit then localizes to the nucleus via the Golgi apparatus and endoplasmic reticulum (ER). In the case of the cytolethal distending toxin (CDT), the A subunit utilizes its type I DNase activity to create double-stranded breaks in host cell chromosomal DNA, activating the DNA damage response and irreversibly arresting the cell in the G_2_/M phase of the cell cycle to cause apoptosis; (**C**) AB_5_ toxins. The holotoxins bind to specific host cell receptors to become endocytosed and undergo retrograde trafficking via early endosomes to the ER through the Golgi apparatus. At the ER, the holotoxin is cleaved, allowing the A subunit to elicit its cytotoxic effects. The A subunit of AB_5_ toxins can target G proteins and adenylyl cyclase, which will increase intracellular cAMP levels and lead to fluid secretion and diarrhea (a). The A subunit also targets ER chaperone proteins, leading to an accumulation of unfolded proteins in the ER to activate the ER stress response and cause apoptosis (b). Lastly, A subunits target residues in 28S rRNA to inhibit protein synthesis, initiate a ribotoxic stress response, and cause apoptosis of host cells (c). Created in BioRender.com (Aoki, S., K. Shteyn, and R. Marien, *BioRender.* Toronto, ON, Canada, Accessed on 4 August 2021).

AB toxins interfere with internal cellular function and are comprised of two-component protein complexes of one active (A) subunit for protein–protein interactions and host cell post-translational modifications and one binding (B) subunit for target recognition and specificity (Figure 1). The general AB toxin mode of action begins with receptor-mediated endocytosis via the binding of the B subunit to host target receptors, causing a conformational change by which the A subunit can enter the designated cell via endosomes. The holotoxins are typically trafficked to the endoplasmic reticulum (ER), where the A and B subunits are cleaved and the A subunit translocates to the host cell cytosol or nucleus to elicit cytotoxic effects [68,69]. Botulinum neurotoxins produced by *Clostridium botulinum* of the *Clostridiaceae* family are possibly the most toxic AB toxins to humans, causing symptoms ranging from slurred speech and blurred vision to muscle weakness and paralysis. Botulinum neurotoxins are comprised of a heavy and a light chain, acting as a zinc-dependent metalloprotease. The heavy chain serves as the B subunit, binding to specific gangliosides and vesicle-associated proteins within the presynaptic vesicles, translocating the light chain (A subunit) into the cytoplasm [70]. The toxin is then free to cleave specific proteins in soluble *N*-ethylmaleimide–sensitive factor-activating protein receptor (SNARE) complexes [70]. This blocks the release of acetylcholine from presynaptic nerve endings and inhibits neurotransmitter-containing vesicles from fusing to presynaptic membranes to cause flaccid paralysis [68]. 

### 2.2. AB_2_ Toxins

AB_2_ toxins, such as CTD (cytolethal distending toxin), contain three subunits—A, B, and C. The CTD holotoxin was first discovered in 1987 by researchers Johnson and Lior and was found to target a variety of mammalian cell types [71]. CTD pathogenesis of *Campylobacter*, *Salmonella enterica*, *E. coli*, and *Shigella dysenteriae* begins with the binding of CtdA and CtdC to host cell ganglioside receptors or to membrane lipid raft microdomains [72]. Receptor binding initiates the internalization of CtdB, which then becomes endocytosed via cytoskeletal rearrangement. The holotoxin may also enter cells via outer membrane vesicles. CtdB then undergoes retrograde transport from the Golgi complex to the ER to localize at the nucleus. CtdB utilizes its type I DNase activity to generate double-stranded breaks in host chromosomal DNA, thus inducing the cellular DNA damage response [72]. Dividing cell populations become irreversibly arrested in the G_2_/M phase of the cell cycle and are thus prevented from entering mitosis, ultimately leading to the induction of programed cell death. Besides stimulating apoptosis, CTD is also responsible for inducing inflammation in the intestines via secretion of IL-8 [73,74], and intestinal epithelial cell production of CXCL8, a chemokine responsible for recruiting polymorphonuclear neutrophils (PMNs) [75]. The translocation of PMN cells across the intestinal epithelium increases the permeability of the epithelia and leads to a leakage of fluid into the lumen to cause watery diarrhea. CTD regulation is closely linked to the cell cycle as well as by themetalloenzyme, LuxS [76].

### 2.3. AB_5_ Toxins

**Figure 2:**
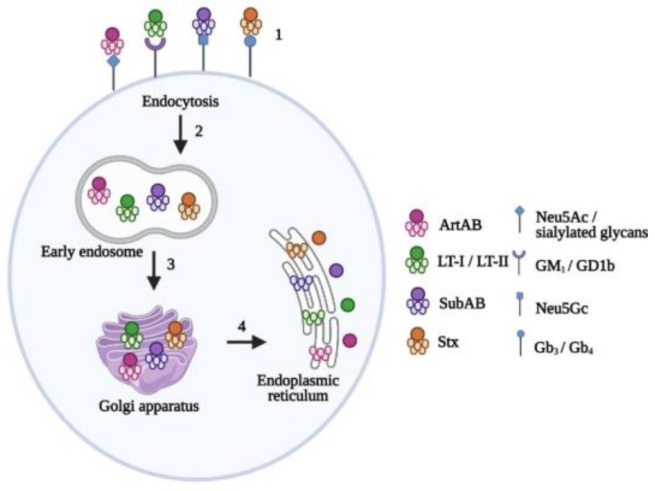
Retrograde trafficking of AB_5_ toxins. (1) Holotoxins bind to target receptors on host cells via the B subunit; (2) binding of the B subunit triggers endocytosis of the toxin, forming early endosomes (3) for transport to the Golgi apparatus; (4) toxins migrate to the endoplasmic reticulum, where the B pentamer subunit is cleaved from the A subunit to allow for downstream cytotoxic effects in the cytosol. LT-I/LT-II: heat-labile enterotoxins; SubAB: subtilase toxin; Stx: Shiga toxin; Neu5Ac: N-acetylneuraminic acid; GM_1_/GD1b: gangliosides; Neu5Gc: α-2-3-linked *N*-glycolylneuraminic acid; Gb_3_:globotriaosylceramide; Gb_4_: globoside. Created in BioRender.com.

AB_5_ toxins have similar structures to AB and AB_2_ toxins, with a composition of six proteins and two subunits. The A subunit is considered the catalytic component and contains two domains (A1 and A2) that are linked by a disulfide bond [77]. A1 is responsible for eliciting toxicity while A2 is non-covalently linked to the B subunit via a central pore. The A subunit is cleaved into its two domains once at the ER of the host cell, allowing A1 to cleave its target molecule [77]. The B subunit of AB_5_ toxins contains five proteins that are either identical or similar in structure and form a pentameric ring. The B subunits are responsible for recognizing and binding specific glycan receptors on host cells to mediate the internalization of the A subunit [77]. AB_5_ toxins utilize a retrograde trafficking pathway like the AB_2_ toxins to reach the cytosol of target cells (Figure 2). AB_5_ toxins are further categorized into four families based on the sequence and catalytic activity of the A subunit. 

## 3. AB_5_ Toxin Families

### 3.1. Pertussis Toxin

**Figure 3:**
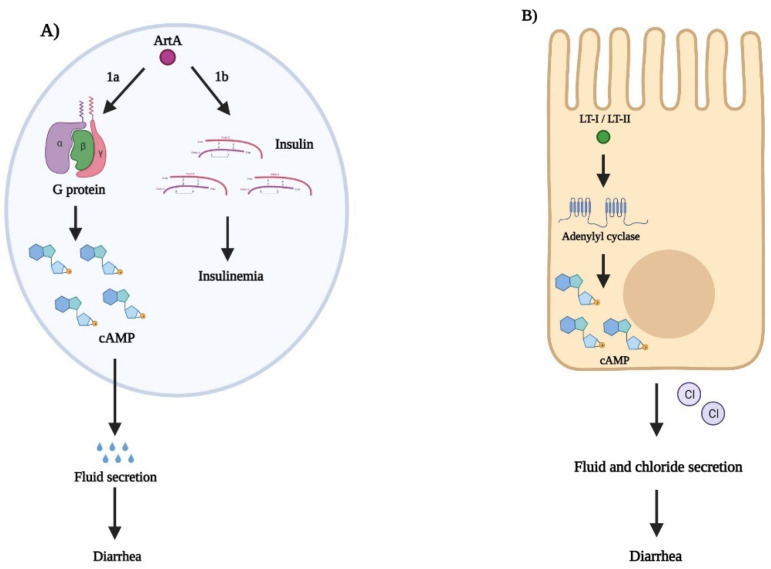

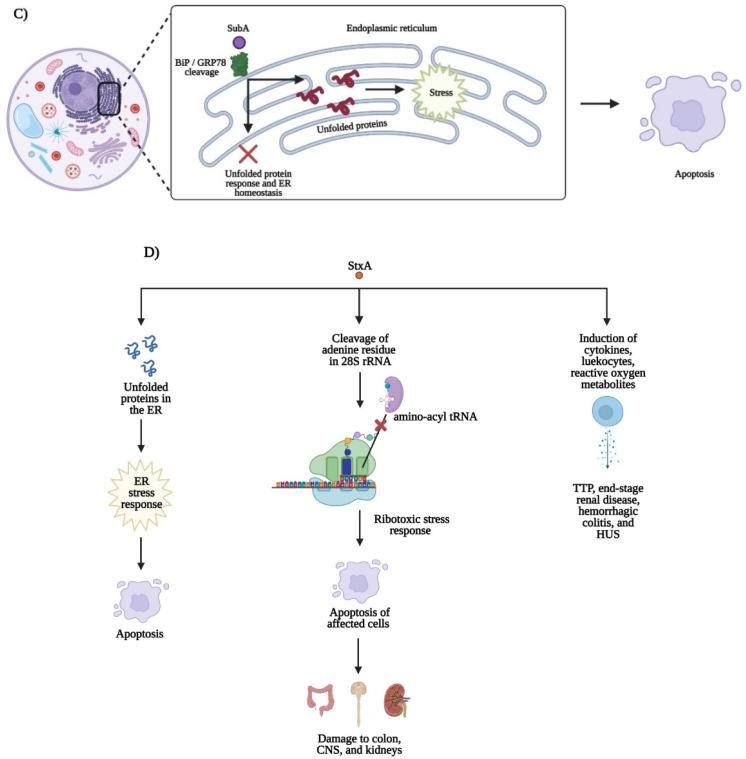
Modes of action of selection toxins from AB_5_ toxin families. (**A**) Pertussis toxin family: ArtA acts as an ADP ribosyltransferase and, after cleavage from ArtB at the endoplasmic reticulum (ER), targets α-subunits of G proteins in the host cell cytosol (1a), leading to an increase of intracellular cAMP levels. The increase in cAMP concentration affects fluid secretion pathways and causes diarrhea. ArtA can also elevate insulin secretion, causing insulinemia (1b); (**B**) Cholera toxin family: LT-I/II targets and activates adenylyl cyclase in epithelial cells, causing an increase in intracellular cAMP concentrations. Elevated cAMP levels stimulate PKA-dependent pathways, creating an imbalance of fluid and chloride ions. Secretion of fluid and electrolytes ultimately results in watery diarrhea and severe dehydration; (**C**) Subtilase cytotoxin: Following cleavage from SubB at the ER, SubA targets and cleaves the ER chaperone protein BiP/GRP78, leading to an accumulation of unfolded proteins. The increased concentration of unfolded proteins initiates an ER stress response, but BiP/GRP78 cleavage prevents homeostasis restoration in the ER and inhibits the unfolded protein response. This leads to protein synthesis inhibition and apoptosis; (**D**) Shiga toxin family: Delivery of Stx to the ER leads to an accumulation of unfolded proteins, causing stress in the ER and apoptosis of affected cells. Stx can also cleave an adenine residue of 28S rRNA of the 60S ribosomal subunit, preventing aminoacyl tRNA from binding and stopping protein synthesis. Affected ribosomes undergo a ribotoxic stress response and apoptosis, causing damage to colon, central nervous system (CNS), and kidney cells. Additionally, Stx induces a pro-inflammatory response by signaling cytokines, leukocytes, and the release of reactive oxygen metabolites, which can lead to severe clinical manifestations such as thrombotic thrombocytopenia purpura (TTP), end-stage renal disease, hemorrhagic colitis, and hemolytic uremic syndrome (HUS). Created in BioRender.com.

The first of four AB_5_ toxin families is the pertussis toxin family. Pertussis toxin (Ptx) is secreted by *Bordetella pertussis*, a member of the *Alcaligenaceae* family, and is the causative agent of whooping cough. *S. enterica*, an enteric pathogen, secretes a toxin similar to Ptx termed ArtAB, which was first discovered in S. enterica serovar Typhimurium DT104 by Saitoh et al. in 2005 [78]. ArtAB is an ADP-ribosylating toxin encoded within various prophages, such as PhInv-1b [19] and Gifsy-1 [20]. ArtAB is categorized as a member of the pertussis toxin family because of the homology between the A subunits of Art and Ptx. ArtA contains conserved amino acid residues—Arg9 and Cys41—for NAD^+^ binding, and His35 and Glu29 for catalytic activity [79]. ArtB mediates endocytosis of ArtA by recognizing and binding to specific glycan receptors on host cells (Figure 3). ArtB is unusual in that it possesses cytotoxic properties by signaling chemokines and cytokines to provoke a strong inflammatory response [48]. The A1 domain interferes with signal transduction once ArtA is endocytosed by targeting α subunits of the heterotrimeric guanine nucleotide-binding proteins (G proteins) in the cytosol, thus acting as an ADP ribosyltransferase [80]. The inhibition of G protein signaling leads to an accumulation of intracellular cAMP levels and to diarrhea by affecting fluid secretion pathways [46]. The effects of ArtA can also result in insulinemia by stimulating insulin secretion and has been shown to result in premature death in neonatal mice following intraperitoneal injection [46]. ArtAB is thought to be regulated by reactive oxygen species like hydrogen peroxide that is released from leukocytes or macrophages under oxidative stress [80]. This may, in turn, induce prophage synthesis, and therefore, ArtAB synthesis.

Extra-intestinal pathogenic *E. coli* (ExPEC), a member of the Enterobacteriaceae family, also contains a pertussis-like toxin (EcPlt), which shares 50–70% identity with Salmonella ArtA proteins [51]. ExPEC is the most common human Gram-negative pathogen and is the cause of several non-intestinal clinical diseases. ExPEC affects people of all ages and is the leading cause of UTIs and adult bacteremia and acquires several antibiotic resistance genes [81]. Inactivation of EcPlt occurs when the NAD^+^ site is blocked by the oxidized state of the A subunit C-terminus, causing the lobe to close, but becomes active once the NAD^+^ site is exposed [51]. Active EcPlt modifies host G_α_ proteins by targeting lysine and asparagine residues, unlike the pertussis toxin which targets a cysteine residue [51]. As of now, little information is known regarding pathogenesis and disease outcomes when EcPlt is secreted in host cells, other than the development of urinary tract infections caused by EcPlt-producing *E. coli*.

### 3.2. Cholera Toxin

The second AB_5_ toxin family is the cholera toxin (CT). Enterotoxigenic *E. coli* (ETEC) secretes toxins similar to that of CT, termed heat-labile enterotoxins (LT-I and LT-II). ETEC colonizes the small bowel mucosal surface and is responsible for causing watery diarrhea, especially in children in developing countries, and is the leading cause of traveler’s diarrhea [82]. ETEC is capable of infecting both humans and other animals and is differentiated from other diarrheagenic *E. coli* by specific virulence factors, such as colonization factors and the LT-I and LT-II enterotoxins. Although these enterotoxins share a homologous structure to CT, the B subunits of LT-I and LT-II have a broader host receptor binding range [83]. LT-I and LT-II are encoded by the *eltAB* operon on plasmid p666. The B subunits bind to lipopolysaccharide (LPS), GM_1_, and GD1b receptors on epithelial cells to initiate cellular pathogenesis and trigger endocytosis (Figure 3) [83]. The A subunit is then cleaved into A1 and A2 domains at the endoplasmic reticulum. The A1 domain activates adenylyl cyclase to elevate internal epithelial cell cAMP levels for the activation of PKA-dependent pathways [83]. PKA-dependent pathways affect fluid secretion in the host by creating an imbalance of water and electrolytes, such as by inhibiting Na^+^ absorption and stimulating secretion of Cl^−^ [84]. This imbalance of water and electrolytes causes the host to have severe watery diarrhea and dehydration while, in turn, aiding ETEC adherence and colonization to intestinal epithelial cells [83]. Positive regulation of LT secretion occurs in the presence of glucose with low cAMP concentrations, while the absence of glucose will raise cytoplasmic cAMP levels and activate CRP binding, resulting in the negative regulation of LT-I and LT-II [85].

Outside of LT toxins, two additional cholera-like toxins have been identified in recent years in *E. coli* and *Citrobacter freundii*, termed EcxAB and CfxAB. EcxB shares a 63% sequence identity with CtxB, and both EcxB and CfxB share structural homology with the B subunit of the cholera toxin, containing six antiparallel beta-strands and two alpha-helices [55]. The B subunit is responsible for binding to ganglioside GM_1_ receptors and CfxB can also bind to type-II antigens of blood group A in addition to GM_1_ [55]. EcxA contains a zinc-binding motif and two disulfide bond-linked domains between cysteine residues 208 and 271, while the A2 domain contains an 8-amino acid redox-switch-linked helix [55]. The A subunit of Ecx is unusual in comparison to other cholera-like toxins due to its function as a metzincin-type metalloprotease—not an ADP-ribosyltransferase—and is thought to target extracellular matrix proteins for degradation. The regulation and overall effect of EcxAB and CfxAB secretion in the host is not yet known. However, the CfxAB toxin family was first isolated in Brazil from children with clinical diarrhea, suggesting a role in causing diarrhea, but culture supernatants containing Ecx and Cfx showed low cytotoxicity in Chinese hamster ovary (CHO) cell and suckling mouse assays [86].

### 3.3. Subtilase Cytotoxin

The subtilase toxin (SubAB) is a relatively new AB_5_ toxin family member, first discovered in 1998 in a O113:H21 STEC strain-related hemolytic uremic syndrome (HUS) outbreak in South Australia [57,87]. SubAB is encoded by subA and subB within plasmid pO113 in more than 30 STEC strains, primarily in those that are locus of enterocyte effacement (LEE)-negative [58,88,89]. SubA shares similarities with serine proteases of the subtilase family, including conserved aspartic acid, histidine, and serine amino acid residues [57]. SubB shares a 50% homology with proteins from *Salmonella typhi* and a 56% homology with exported proteins from *Yersinia pestis* [57], suggesting SubB and *Y. pestis* proteins may be both structurally and functionally related. SubB first binds to host glycoprotein sialic acid, specifically α-2-3-linked N-glycolylneuraminic acid (Neu5Gc), to initiate uptake of SubA via a clathrin-dependent retrograde trafficking pathway through the Golgi complex to the endoplasmic reticulum (Figure 3) [59]. The A subunit cleaves the ER chaperone protein, BiP/GRP78, leading to an accumulation of unfolded proteins in the ER [58]. This accumulation of unfolded proteins triggers ER stress-signaling pathways, but the cleavage of BiP/GRP78 blocks the unfolded protein response and prevents ER homeostasis to result in apoptosis and the inhibition of protein synthesis. The expression of SubAB is regulated by global regulator proteins Hfq and H-NS [90], and secretion of SubAB can lead to vacuolation and apoptosis of Vero (kidney epithelial) cells by cytochrome c release and caspase activation [60]. SubAB intraperitoneal injection in mice led to striking resemblances with STEC-associated HUS in humans, as well as to leukocytosis, thrombocytopenia, and renal cell and liver damage [91]. The contribution of SubAB to human HUS pathophysiology is currently unclear but is thought to act in synergy with Shiga toxins to enhance clinical manifestations of STEC infections [92,93].

### 3.4. Shiga Toxins

The fourth and final AB_5_ toxin family is arguably the one that causes more severe clinical manifestations throughout infection. The prototypical Shiga toxins, StxA/B, were first discovered in *S. dysenteriae* type I—the causative agent of bacillary dysentery—by Japanese scientist Dr. Kiyoshi Shiga [94,95]. Shiga toxins were thought to be unique to *S. dysenteriae* type I for several years before clinical isolates of other *Shigella* species were found to harbor *stx,* including *S. dysenteriae* type 4 [65], *Shigella sonnei* [66,96], and *Shigella flexneri* [97,98]. Shiga toxin-encoding genes are found on lambdoid bacteriophages; however, unlike the Stx-converting phages in *E. coli,* phages in *S. dysenteriae* are not intact, possibly due to transposition and recombination events that led to the loss of phage gene function [99,100]. The pathogenesis of Stx starts with the B subunit directly binding to eukaryotic cell globotriaosylceramide (Gb_3_) receptors, primarily located on endothelial cells, to initiate internalization and retrograde trafficking to the ER and cytosol [101]. Delivery of StxA to the ER causes an accumulation of unfolded proteins in the ER, thus stimulating an ER stress response that results in apoptosis of host cells [102]. The StxA1 domain also cleaves an adenine residue of 28S rRNA of the 60S ribosomal subunit to cause an inhibition of aminoacyl-tRNA binding, and subsequently, an inhibition of host protein synthesis [103,104]. The damaged ribosomes activate the ribotoxic stress response in affected cells and stimulate a pro-inflammatory and pro-apoptotic signaling cascade that involves the activation of mitogen-activated protein kinases (MAPKs), jun-N-terminal kinases (JNKs), p38, and extracellular-receptor kinases (ERKs) [105,106]. The pro-inflammatory response results in microvascular endothelial cell damage in the kidneys, colon, and central nervous system. Additionally, Shiga toxins can alter host cell signaling and reduce the tight junction integrity along the gastrointestinal tract, ultimately leading to electrolyte loss and watery diarrhea [103]. The regulation of StxA/B are under the control of an authentic iron-regulated promoter. 

Shiga toxins are considered the cardinal virulence factors for various *E. coli* (STEC) serovars, the first of which was identified from two separate outbreaks of hemorrhagic colitis (HC) in Oregon and Michigan in 1982 [107,108]. The outbreak, caused by *E. coli* O157:H7, was associated with the consumption of undercooked beef at a national fast-food restaurant. The outbreak affected at least 47 individuals, with illness characterized by little-to-no fever, severe abdominal cramping, and bloody diarrhea. Not all STEC strains are human pathogens, but do express at least one Shiga toxin, similar to that of the prototypical Shiga toxin identified in *S. dysenteriae* type 1, in that the A subunit inhibits eukaryotic protein synthesis via removal of an adenine residue from 28S rRNA and can cause direct cell damage, apoptosis, and anemia to the host (Figure 3) [103]. The B subunit binds Gb_3_ receptors for toxin internalization while in the intestine microvasculature and, once gaining access to the bloodstream, stimulates a pro-inflammatory response [109]. In addition, Shiga toxins can cause severe intoxication symptoms, such as end-stage renal disease, hemorrhagic colitis, and HUS [103]. Severe clinical manifestations are primarily a result of the local induction of pro-inflammatory cytokines, leukocytes, and the generation of reactive oxygen metabolites that damage intestinal mucosal tissue and instigate hemolysis [109,110]. Glomerular thrombi and epithelial cell damage lower the kidney filtration rate, ultimately resulting in renal failure. Regulation of *stx_1_* and *stx_2_* occurs via a number of processes and proteins, including the activation of the lytic cycle during stress conditions, bacterial cell lysis, RecA, iron-regulated promoters, and cleavage of the cI promoter [111]. Understanding the underlying mechanisms and pathogenesis of Shiga toxins is important to develop effective prevention and treatment strategies.

To colonize and elicit infection within a host, pathogenic *E. coli* must compete with normal flora for both space and nutrients. Factors such as antibiotics and diets can influence shifts in intestinal microbiota and possibly affect the severity of STEC infections [112]. For example, two-week old pigs challenged with the O157:H7 strain 86-24 had different intestinal microbiota in ligated ileal loops. Pigs with no attachment and effacing (A/E) lesions experienced an increase in the probiotic bacteria *Veillonella* *caviae* and *Bacteroides*, and a decrease in *Clostridium* spp., suggesting a role for these normal flora in A/E lesion development [113]. The release of soluble factors and metabolites by *Bacteroides thetaiotamicron* also influences the virulence determination of enterohemorrhagic *E. coli* (EHEC). EHEC are a subset of STEC responsible for causing, attaching, and effacing lesions and severe disease in humans, such as hemorrhagic colitis, hemolytic uremic syndrome, and thrombotic thrombocytopenic purpura. Soluble factors repress EHEC transcriptional Stx2 synthesis *via* inhibition of the RecA-mediated SOS response [114], while proteases cleave type III secretion translocation and enhance effector translocation into host cells [115]. Fucose released from the intestinal mucus by *B. thetaiotamicron* decreases STEC LEE gene expression [116], yet it is enhanced under gluconeogenic conditions [117], while depletion of vitamin B_12_ (a modulator of Stx2 production) in the gut by *B. thetaiotamicron* inhibits Stx2 production [118]. EHEC can also take environmental cues as signals to direct the expression of certain virulence genes. The fermentation of sugars by colonic normal flora, primarily *Bacteroides* and *Firmicutes*, results in the production of short-chain fatty acids (SCFAs), such as acetate, succinate, and butyrate [119]. Concentrations of SCFAs vary throughout the gastrointestinal (GI) tract, with lower concentrations in the upper GI tract and higher in the proximal colon, which EHEC utilizes as an environmental signal. Succinate produced by *Bacteroides* spp. is sensed by EHEC via the transcription factor Cra to enhance virulence gene expression and A/E lesion formation [120]. Butyrate at 1.25–40 mM induces EHEC strain Sakai type III secretion and enhances cell adherence and A/E lesion gene expression via a signal cascade involving a leucine-responsive regulatory (Lrp) protein [121]. Butyrate is also known to upregulate the receptor for Stx, Gb_3_, in renal tissue and the colonic epithelium [122], and increases adhesion and microcolony formation to epithelial cells [123]. Additionally, secreted molecules, such as bacteriocins (proteinaceous toxins that target closely related bacteria), released by commensal *E. coli* strains, induce the SOS response and stimulate phage induction and Stx release via DNA damage [124,125,126]. Human-derived microbiome metabolites, such as 4-methyl benzoic acid, 3,4-dimethyl benzoic acid, hexanoic acid, and heptanoic acid, may also promote HUS pathogenesis by preferentially enhancing the motility of *E. coli* O157:H7 via the induction of flagellin expression, although Stx expression and *E. coli* O157:H7 colonization are not affected [127].

## 4. Evolution, Lineage, and Stx Variants of *E. coli* O157:H7

*E. coli* are very diverse in both genetics and pathogenicity. Pathogenic *E. coli* strains can be categorized by pathotype, phylogroup, seropathotype, lineage, and clade. There are six major *E. coli* pathotypes responsible for causing diarrhea in humans: EPEC, STEC/EHEC, EAEC, ETEC, enteroinvasive *E. coli* (EIEC), and diffusely adherent *E. coli* (DAEC). The genomes of *E. coli* include a conserved set of genes (core genome) and a flexible gene pool, which largely contributes to pathogenesis, mainly via mobile genetic elements. Both genotypic and phenotypic traits are used to designate a pathotype to *E. coli* isolates and the genome plasticity contributes to the emerging pathogenic *E. coli* hybrids, as seen in the 2011 German outbreak of STEC O104:H4, an enteroaggregative hemorrhagic *E. coli* strain.

EPEC was the first diarrheagenic *E. coli* pathotype identified, with the hallmark trait of causing A/E lesions on intestinal epithelial cells. While strains in this pathotype do not produce Shiga toxins or heat-labile (LT) or heat-stabile (ST) enterotoxins, typical EPEC do have the *E. coli* adherence factor plasmid (pEAF) while atypical EPEC strains do not [128]. Some atypical EPEC strains are more closely related to those of the LEE-positive STEC, as comparative genomic and proteomic analyses indicate STEC O157:H7 evolved from an EPEC O55:H7 ancestor [129]. Clonal lineages of EPEC appeared to have evolved through the independent acquisition of pEAF and LEE [130]. EPEC causes infantile diarrhea and is prevalent in developing countries. The next pathotype, STEC, are defined as *E. coli* strains with the presence of Stx1 and/or Stx2. There have been more than 400 STEC identified, but only a portion are related to causing illness in humans [131]. Of this subset, EHEC causes mild-to-bloody diarrhea and HUS, and was originally described by its association with hemorrhagic colitis and differing traits from EPEC [132]. EHEC pathotype isolates are typically LEE-positive and cause A/E lesions, but some LEE-negative strains are known to cause hemorrhagic colitis and HUS as well. The third pathotype is EIEC, which are intracellular pathogens responsible for causing bacillary dysentery similar to shigellosis. EIEC strains were discovered about 50 years after *S. dysenteriae* and share several biochemical, genetic, and pathogenic traits with *Shigella* species [133]. Multi-locus and whole-genome analyses found that *Shigella* and EIEC form a single pathotype within *E. coli*, with *Shigella* having more similarities to *E. coli* K12 than O157:H7 [134]. EIEC are believed to have emerged from commensal *E. coli* via the acquisition of invasion plasmid pINV [135]. Serotypes within the EIEC pathotype are defined by their O-antigen pattern, with some being identical or similar to those of Shigella, with it proving difficult to differentiate between EIEC and *Shigella* isolates [136]. The EAEC pathotype was identified by comparing the adherence patterns of more than 500 isolates in 1987 [137], with the further classification of strains into typical (contains aggR and colonizes the small bowel) or atypical (lacks *aggR* and colonizes the small bowel and colon) categories. Strains in this pathotype cause persistent diarrhea in children as well as traveler’s diarrhea, with perhaps the most well-known isolate from this pathotype being O104:H4, the STEC hybrid strain responsible for the 2011 German outbreak [138]. ETEC are a very diverse pathotype with isolates represented in all phylogroups causing significant mortality in children in developing countries, and with an adverse effect on the swine industry as well [128]. ETEC pathotype strains express LT or ST enterotoxins and various colonization factors and display a wide distribution of O- and H-antigens. A possible reason ETEC strains are pathogenic is due to the acquisition of plasmid-borne toxins and virulence factors, although these profiles vary among geographic regions [139,140]. The sixth pathotype, DAEC, is defined by the diffuse adherence pattern on HEp-2 and HeLa cells, but they are difficult to identify and classify [141]. This pathotype is associated with persistent watery diarrhea in children and carriage of DAEC could contribute to the development of chronic inflammatory intestinal disease in adults [142]. 

Evolutionary and phylogenetic models were proposed after the 1982 outbreak to outline the emergence of the rare serotype, *E. coli* O157:H7. Clermont et al. designed a triplex-PCR method consisting of two gene markers (*chuA, yjaA*) and an anonymous DNA fragment (TSPE4.C2) to sort *E. coli* strains into phylogenetic groups based on marker distribution [143]. The distribution grouped E. coli strains into four phylogroups: A, B1, B2, and D, and multi-locus sequence typing confirmed 80–85% of the phylogroup classifications [144]. Clermont et al. added an additional gene target in 2013, *arpA*, to further classify *E. coli* into phylogroups A, B1, B2, C, D, E, F, and clade I with 95% accuracy [145], and in 2019 added an intermediate group between B2 and F, termed phylogroup G, that is common in livestock and poultry and has extensive antibiotic resistance [146]. *E. coli* strains belonging to phylogroup A are predominant in humans, B1 strains are predominant in animals [147], with phylogroup C strains closely related to those in B1 [148]. Virulent extraintestinal *E. coli* are primarily categorized into phylogroups B2, D, and F, while several commensal strains are grouped as A or D [145,149,150,151]. 

STEC strains can further be categorized into one of five seropathotypes (A to E) by their association with human disease, outbreaks, and HUS [152]. Karmali et al. defined the seropathotypes with a decreasing rank of pathogenicity, in order to further understand the differences between STEC serotypes in terms of virulence, with differences associated with the presence or absence of pathogenicity islands to aid in identifying appropriate DNA targets for the detection of STEC that pose a significant risk to human health [152]. Seropathotype A strains are classified as those with a strong association with HC and HUS; seropathotype B strains are also associated with HC and HUS but to a lesser extent than those in A; strains grouped in seropathotype C are correlated with sporadic HUS cases; seropathotype D are strains with diarrhea, but not with outbreaks or HUS; and strains in seropathotype E are isolated from non-human sources and are not associated with human illness. Karmali et al. studied the distribution of *E. coli* strain EDL933 O-island 122 (OI-122) in 70 STEC strains, of which a complete OI-122 and the presence of eae were found to be strongly associated with seropathotypes related to epidemic disease (A and B) and those related to HUS (A, B, and C), and of serotypes represented by more than one strain, each strain had identical OI-122 and eae patterns [152]. Additionally, strains with an incomplete OI-122 progressively increased from seropathotypes A to E. Serotypes O157:H7 and O157:NM were found to correspond to seropathotype A; O26:H11, O103:H2, O145:NM, and O121:H19 to seropathotype B; O91:H21 and O113:H21 to seropathotype C; and O146:H21 and O132:NM to seropathotype D [152]. Furthermore, Toma et al. found that strains in seropathotype A have a unique adhesin profile, and that adhesin lpfA_O113_ was absent in seropathotype A but present in all other seropathotypes, as was adhesin iha [153]. Taken together, these studies suggest there is a link between serotypes, seropathotypes, pathogenicity islands, and clonal groups consistent with the acquisition of genetic elements. However, not all STEC have been well characterized and the emergence of hybrid strains challenge the seropathotype classification.

Evolutionary models proposed that *E. coli* O157:H7 evolved in a stepwise manner from an enteropathogenic strain of O55:H7, which lacks Shiga toxin genes but expresses β-glucuronidase and ferments the sugar alcohol sorbitol [154]. The first divergence of strain O55:H7 involved the acquisition of stx_2_ via phage transduction, followed by a lateral transfer of an rfbE-like region to give rise to O157 antigens [155]. The immediate ancestor of the O157:H7 serotype lost sorbitol fermentation and β-glucuronidase activity, followed by stx_1_ acquisition through bacteriophage transduction [154] to become an emerging human pathogen. This model by Feng et al. is based on three assumptions: loss of function of metabolic genes exceeds that of gain of function, lateral transfer is used to acquire gain of function genes, and the preferred model uses the least number of total steps for emergence. Additional models have been proposed in more recent years using single nucleotide polymorphisms (SNPs) and sequencing data to demonstrate the variation in presence of stx_1_- and stx_2_-converting phages [156,157,158]. The newer models also incorporate the multiple acquisitions and losses of the phages in both O55 and O157 serotypes, thus demonstrating the unreliability of stx_1_ and stx_2_ phages to serve as evolutionary markers [156,157].

The *E. coli* O157:H7 evolutionary relation and strain origin can additionally be observed through lineage and clade classification. There are currently three lineages (I, II, and the intermediate lineage I/II), seven sub-lineages (Ia, Ib, Ic, IIa, IIb, IIc, and I/II), and nine distinct phylogenetic clades associated with human disease outbreaks [159]. Grouping into lineages is typically performed using octamer-based genome scanning (OBGS), lineage specific polymorphism assays (LSPA-6), SNP genotyping, comparative genomic hybridization, restriction fragment length polymorphism analysis, or pulsed-filed gel electrophoresis (PFGE). OBGS analysis was first utilized in 1999 to establish the divergence of O157:H7 into two separate lineages, I and II, with unique ecological characteristics [160]. Further analysis using the six-marker test, LSPA-6, in 2004 added a third lineage—the intermediate I/II [161]. It is believed that the A5 O157:H7 β-glucuronidase-positive sorbitol-negative ancestor gave rise to lineage II, which is commonly associated with animals (particularly ruminants), and often these strains have decreased stx_1_ expression and are considered as less pathogenic [162]. Lineages I and I/II are thought to have independently stemmed from a stx_2c_ phage-containing strain. Lineage I strains are often linked to human infections—possibly due to higher Stx2 expression compared to other lineages—indicating unique virulence expression patterns for enhanced human colonization [159]. Meanwhile, lineage I/II strains possess characteristics of lineage I and/or lineage II, with several of these strains colonizing in cattle. The differential expression of stx_2_ among lineages provides insight on why certain strains are more pathogenic than others amongst lineages. This ideology can also be applied to clades originally differentiated based on SNP genotyping and associated with severe disease like HUS, such as clades 6 and 8 [159,163].

Shiga toxins secreted by *E. coli* are diverse, with variations in amino acid sequence and cytotoxicity. The two Shiga toxin subtypes share a 56% amino acid identity [164] and occasionally have biased distribution in host specificity. The Stx1 family has only three known variants (a, c, d), while Stx2 is more heterogenous with seven allelic types (a-g) [165]. Stx1 was the first Shiga toxin identified in bacterial species outside of *S. dysenteriae* type I [166], but it is nearly identical to the prototypical Shiga toxin, with only three nucleotide changes resulting in one amino acid difference [167]. Stx1 variants are considered less cytotoxic than those of Stx2, based on the higher catalytic activity and affinity for yeast and mammalian ribosomes of the A1-Stx2 subunit in comparison to that of Stx1 [168]. Additionally, Stx2 is correlated with the clinical severity of STEC infections more so than Stx1 [169,170], as Stx1 alleles are often identified from asymptomatic or non-severe diarrheic patients. A phylogenetic study of *E. coli* O157:H7 strains by Dallman et al. revealed that Stx2aϕ acquisition was a more recent event in comparison to other Stx variants and appeared to be conserved once incorporated into strain populations [162]. The same study also found that stx_2a_ was necessary for HUS development in a cohort of 500 *E. coli* O157:H7 clinical cases [162]. 

Stx2 variants have been isolated from multiple sources, both human and non-human. Stx2a, Stx2c, and Stx2d are closer in sequence, isolated from human infection sources, and are frequently associated with severe-infection clinical manifestations, such as hemorrhagic colitis and HUS [171,172]. These variants are often isolated from bovines as well, and are the major Shiga toxin subtypes present in O157:H7 strains. Stx2d is biologically different from other Stx variants in that it is activatable when incubated in mouse or human intestinal mucus, increasing its Vero cell cytotoxicity by 10–1000-fold [173]. Stx2d cytotoxicity is also enhanced by the proteolytic enzyme elastase, which acts by cleaving two amino acids within the A2 subunit at the C-terminal, therefore altering the mobility and isoelectric point of the subunit [174]. On the other hand, variants Stx2b, Stx2e, Stx2f, and Stx2g are distantly related and are more frequently found in non-human sources harboring STEC strains, such as deer, pig, and cattle, and elicit less serious disease than other Stx2 variants [175]. Stx2e is associated with causing edema disease in neonatal piglets [176] and has occasionally been identified in asymptomatic human and fresh produce isolates, although not as often as Stx2a or Stx2d alleles [177]. Unlike the other subtypes, Stx2e binds Gb_4_ receptors in preference to Gb_3_. Stx2f is frequently isolated from pigeons and other birds [178], but has also been isolated from diarrheic patients [179,180]. Three new Stx2 subtypes have been discovered within the last five years: Stx2h, Stx2i, and Stx2k. Stx2h was identified in a 2018 study of pathogenic *E. coli* population isolates in wild animals that were separated from human activity (natural habitat) in China [181]. Based on sequencing, Stx2h was detected in four of six (66.7%) wild marmot *E. coli* O102:H18 isolates, but has not yet been detected in any other animal or human strains from China. The Stx2i variant was identified in 2016 in a microarray study examining expedited alternatives for STEC characterization [182]. Forty-seven *E. coli* isolates from 39 food samples were examined in the study, of which an O:H25 strain isolated from shrimp contained an unnamed Stx2 variant allele sequence—the provisional Stx2i. Lastly, Stx2k was detected in multiple non-O157 serotypes isolated from raw meat, goats, pigs, and diarrheal patients across China in 2019 [175]. This variant appears to be similar in receptor-binding preference, acid tolerance, and thermostability to that of Stx2a, yet appears to be less toxic in Vero cell assays [183]. Stx2k was initially assigned as a new Stx2e variant produced by strain STEC388 because it was isolated from healthy pigs [184], but sequencing characterization revealed otherwise [175]. 

## 5. Stx Pathogenesis and Disease

Shiga toxin pathogenesis is a multistep process, incorporating the interactions between host and bacterial factors. Human pathogenesis typically begins with the oral ingestion of EHEC via contaminated food or drink, as observed in the 1982 outbreak. The annual global incidence of acute STEC infections was estimated to be around 2.8 million in 2014 [185]. Although the incidence is lower than other common foodborne pathogens, EHEC can lead to more severe infections and complications than other pathogens of this nature and, because of HUS, is the leading cause of acute renal failure in children in developed countries [186,187]. The key virulence determinants of STEC early on in human infection include surviving passage through the harsh environment of the stomach through the expression of acid resistance systems, the ability to compete with the gut microbiota for colonization, and adherence to host intestinal epithelial cells. To elicit cytotoxic effects, Shiga toxins produced in the lumen must be delivered to underlying tissues and organs via bloodstream trafficking. Since most STEC are considered as non-invasive, the translocation of Stx and other virulence factors may occur through the generation of systemic sequelae in the intestines [67]. This process may involve lesion formation and erosion within the intestinal mucosal barrier and lamina propria, passage through gaps between adjacent epithelial cells, or passage through intact epithelial cells without causing cellular disruption [188]. Once in the lamina propria, Shiga toxins can trigger colonic blood vessel damage and inflammation by inducing the expression of chemokines and inflammatory T-cell subsets via intestinal epithelial cell-secreted immunomodulatory molecules [189,190,191]. The induction of pro-inflammatory molecules by Stx1 and Stx2, such as TNFα and IL-1β, may sensitize endothelial cells to the toxins by upregulating Gb_3_ receptor expression, thus contributing to vascular damage in the gut and kidneys [192,193]. Most Shiga toxins do not circulate in the blood in free form, but instead bind to red blood cells [194], leukocytes [195], neutrophils [196], monocytes [197], and platelets [198]. Shiga toxins bound to red and white blood cells trigger the cells to shed microvesicles, which are pro-inflammatory, pro-thrombotic, and allow the toxins to evade the immune system and degradation, and therefore, transport the toxins to target cells and tissues [199,200,201]. The main target receptors of Shiga toxins are Gb_3_ or Gb_4_ in kidney, gastrointestinal, and cerebral epithelia [103]. This toxin–cell interaction leads to an increase in cytokine circulation, cell death, and destruction of blood cells and platelets. Human kidneys have high levels of Gb_3_ receptors and are the primary site of renal lesions in HUS patients [202]. In addition, microvesicles bound to Stx1 and Stx2 have been identified in the blood and kidneys of patients infected with EHEC [201]. 

Shiga toxins also damage microvascular endothelial cells within the kidneys and central nervous system [199] and induce several histopathological changes. The lumen of glomerular capillaries narrows with swollen and damaged endothelial cells and the cells detach from the underlying basement membrane—a histopathological hallmark of diarrheic HUS. This leads to local cytokine and chemokine production, secondary coagulation activation, and fibrin synthesis [190,203]. Damaged endothelial cells can also trigger the deposition of platelets in microvascular thrombi, resulting in thrombotic microangiopathy and the destruction of red blood cells in the occlusive capillary lesions [204]. The narrowing of glomerular capillaries reduces the blood flow to the kidneys, decreases the filtration rate, and can ultimately result in renal failure [67,205]. Further renal injury can occur through the tubular necrosis of proximal tubular epithelial cells. Microvascular endothelial cell damage can also affect the brain, pancreas, and myocardium, leading to the development of encephalopathy, diabetes mellitus (particularly in adults), cardiomyopathy, hemorrhagic colitis, and thrombotic thrombocytopenic purpura (TTP) [206]. Specifically, StxB has been shown to promote the development of TTP by stimulating the secretion of von Willebrand factor (vWF) in human umbilical vein endothelial (HUVEC) cells and promoting platelet adhesion [207].

In the early stages of HUS, several patients have elevated markers related to tubular injury and necrosis [208], increased polymorphonuclear leukocytes (PMNs) counts [209], and elevated plasma IL-8 levels [210]. Elevated IL-8 concentrations act as a chemoattractant for PMNs, which can release elastase to degrade the extracellular matrix of endothelial cells, partly attributing to their release from the basement membrane [210]. Diarrheic HUS associated with EHEC infections primarily occurs in children, with obesity being an additional susceptibility factor [211], but it is estimated that 5–15% patients with bloody diarrhea will develop HUS and acute renal failure [212]. Studies have shown that there is a correlation between the type of Shiga toxin secreted and the course of infection that ensues [213]. For example, patients have a higher rate of developing HUS when *stx_2_* subtypes are secreted, rather than those of *stx_1_* [214,215], especially *stx_2a_* [216] and *stx_2c_* [171]. STEC expression of *stx_2a_* increases the risk for renal and central nervous system complications as well [169,217]. Matthews et al. found that expression of *stx_2a_* and *stx_2c_* not only causes severe infection in humans but can also lead to higher levels of *E. coli* O157:H7 shedding in cattle [218]. Epidemiological studies of EHEC outbreaks have also found that diarrheic HUS cases predominantly affect women, with 62.1% of cases in a 1999 China *E. coli* O157:H7 outbreak [219] and 58% of cases in the 2011 Germany *E. coli* O104:H4 outbreak occurring in women [220]. Production of such Shiga toxins can be utilized for the detection of STEC during infection. 

## 6. Stx Detection and STEC Therapies

Methods for STEC and *stx* detection include culturing methods, molecular-based approaches, and serological methodologies. One of the more common STEC detection methods includes culturing stool samples on selective and differential media, such as sorbitol-MacConkey (SMAC) agar, CHROMagar^TM^ STEC, Rainbow^®^ Agar O157, or Tryptone Bile X-glucuronide (TBX) agar [221,222]. However, several STEC non-O157 strains are sensitive to potassium tellurite additives in certain recovery and isolation media, with it proving difficult to grow and identify such strains [223]. A combination of both highly selective and inclusive (or less selective) mediums could improve the recovery frequency and identification of non-O157 serotypes [223,224]. The confirmation of STEC isolates can be performed using enzyme immunoassays, like immunochromatographic and microwells, or molecular-based methodologies, like real-time and multiplex PCR, to detect *stx_1_*, *stx_2_*, *eae*, and hemolysin virulence genes [222]. Shiga toxins can also be detected using toxicity assays. The Vero cell cytotoxicity neutralization assay incubates filtered fecal samples with Vero cells, which contain several Gb_3_ receptors, to observe a cytopathic effect from the presence of Shiga toxins. The effects are confirmed to be caused by Shiga toxins by neutralizing the toxins with anti-Stx1 and anti-Stx2 antibodies [225]. A 2016 study evaluated the performance and accuracy of a rapid membrane immunoassay—the Shiga Toxin Quik Chek (STQC)—in comparison to the Vero cell cytotoxicity assay, and found that STQC is able to detect all described Stx subtypes and correlates 100% with the Vero cell assay using clinical samples [226].

Public health often warrants further identification of STEC isolates, especially in outbreak situations, to determine the relatedness of such isolates and to trace back to the source. To do this, pulsed-field gel electrophoresis or sequencing are usually performed. PFGE is often subjective and time-consuming, while sequencing provides a more streamlined identification process and allows researchers to analyze the entire genome of an isolate rather than looking for specific genes. The most efficient way to detect and identify STEC in samples incorporates multiple techniques and methodologies, such as culturing on selective and differential media and utilizing qPCR [213].

Currently there are no effective treatments for STEC infections other than supportive therapies to aid in the prevention of HUS development. The administration of fluids and electrolytes is recommended during vomiting and diarrhea to provide hydration and protect from oligoanuric renal failure in children [227], although if the patient has edema or hypertension, fluid restriction is the recommended course of action to minimize fluid overload [228,229]. Fluid overload could contribute to hypertension, which has been shown to occur in 15% of STEC cases in children [230], of which high blood pressure can further contribute to thrombotic microangiopathic lesion formation [231]. For STEC infections that have progressed to HUS, renal replacement therapy, such as hemodialysis in adult infections and peritoneal dialysis in pediatric infections, and erythrocyte transfusions are recommended [229]. Platelet transfusions are suggested only for patients with severe bleeding or thrombocytopenia [228].

Emerging alternative therapies for STEC infections include vaccines/immunization, phage therapy, probiotics, and natural products. Several STEC vaccines are plant- or bacteria-based or centered around STEC toxins. Orally immunized mice were protected from the challenge of *E. coli* O91:H21 strain B2F1 by using Stx2 toxoid-expressing NT-1 cells from a *Nicotiana tabacum* (tobacco) cell line [232], and oral vaccination of piglets with *stx_2eB_*-transgenic lettuce prior to virulent STEC challenge resulted in the decreased pathogenesis of edema disease associated with Stx2e-producing *E. coli* [233]. Attenuated vaccines of *E. coli* O157:H7 strain 86-24 reduced intestinal colonization in mice with no clinical signs of disease [234], and mice pre-challenged with enteropathogenic *E. coli* developed mild symptoms after EHEC infection but recovered, compared to mice not pre-challenged, resulting in mild to severe symptoms and changes in renal histopathology [235]. *Salmonella-* and *Lactococcus lactis*-based vaccines expressing either Stx2 or EspA protected mice for 70 days after challenge with a lethal dose of EHEC [236] and minimized intestinal colonization and kidney damage [237,238]. Additionally, several STEC vaccine strategies are based solely on toxins, mainly Stx2 due to its association with severe disease symptoms in human infections [239,240,241,242]. Other alternatives, such as toxin intracellular interference [243] or antibody therapy using stx-specific monoclonal antibodies to clear the toxins from the bloodstream [244], are also being evaluated.

The application of bacteriophages, or phage therapy, has recently emerged as having promise as a prophylactic treatment of STEC infections. In vitro application of two T4-like *Myoviridae* exogenous lytic phages, p000v and p000y, prior to lysogen induction, reduced Shiga toxin-phage production in O157:H7 and, therefore, toxin amplification [245]. Two bacteriophages, vB_Eco4M-7 and ECML-117, possess ideal properties for fighting STEC infections, including a lack of genes coding for toxins and lysogenization-specific genes, effective infection of several O157 strains, and rapid lytic development [246], although further studies are needed on these bacteriophages for their use in therapy. Furthermore, a single treatment of a T7-like STEC lytic phage (PHB19) resulted in the survival of all mice infected with STEC strain HB10, irrespective of administration simultaneously with challenge or 3 h prior to or after bacterial challenge [247]. A combination of phage CA933P and probiotics was shown to reduce adherence of O157:H7 to epithelial HEp-2 cells In vitro by 1–2 log CFU [248].

Probiotics are known to compete with bacterial pathogens for nutrients and space in a host and are able to produce antimicrobial substances [249]. Probiotics on their own are not routinely used in managing human STEC infections, although animal studies suggest a role in preventative care. Probiotic application in ruminants reduced fecal shedding of O157 [250,251,252] and reduced morbidity in STEC-infected mice with application before and after infection [253]. The *Saccharomyces cerevisiae* strain CNCM I-3856 directly antagonized *E. coli* O157:H7 in vitro under simulated human gastrointestinal tract conditions, leading to a decrease in bacterial survival, possibly via ethanol production [254], and a down-regulation in Shiga toxin production [255]. In addition, *S. cerevisiae* CNCM I-3856 influenced the gut microbiota via short-chain fatty acid modulation to increase acetate and decrease butyrate production [255,256]. *Lactobacillus* species have demonstrated an inhibitory effect on the growth [257] and adhesion to Caco-2 [258] and HT-29 cells [259] of STEC in vitro. In addition, fractions of *Lactobacillus acidophilus* strain La-5 cell-free spent medium (CFSM) reduced *E. coli* O157:H7 extracellular autoinducer-2 (AI-2) activity in vitro by acting as a quorum-sensing signal inhibitor, and the expression of LEE-encoded genes such as *tir, eaeA, fliC, luxS,* and *espA* was reduced in the presence of 10% La-5 CFSM fraction, but had no significant impact on Shiga toxin production [260]. However, coincubation of *E. coli* O157:H7 with probiotic bacteria, such as *Bifidobacterium, Pediococcus,* and *Lactobacillus*, at sub-lethal concentrations results in the downregulation of *stx_2a_* expression in vitro, possibly due to the low pH achieved by the production of organic acids by the probiotic bacteria [261].

Natural products, such as medicinal plants, extracts, and bacterial products, also have an effect on *E. coli* O157:H7. Essential oils from savory (*Satureja montana*), Spanish oregano, and Chinese cinnamon alter the bacterial membrane integrity and induce intracellular ATP depletion [262]. Furthermore, ellagitannin extracted from *Quercus infectoria* was shown to reduce intestinal O157:H7 colonization and protect infected mice from renal injury [263], while Shiga toxin cytotoxicity was inhibited by fruit extracts of the white carob tree and *Ziziphus mistol* in a Vero cell system [264]. STEC treated with *Quillaja saponaria* bark extracts for 1 h resulted in a reduction of STEC in vitro and damage to the bacterial cell membrane [265], while grape seed extracts inhibited the growth of the top six non-O157 STECs and suppressed quorum sensing [266]. Additionally, ethanolic leaf extracts from medicinal plant *Rhodomyrtus tomentosa* possibly enhances neutrophil activity against enterohemorrhagic *E. coli* and alters the bacterial membrane integrity [267], and a variety of STEC isolates from food, clinical, and veterinary samples were inhibited by sorrel (*Hibiscus sabdariffa*) phenolic extracts in vitro at concentrations of 2.5%, 5%, and 10% [268]. 

The use of antibiotics is strongly discouraged to treat STEC infections due the possibility of antibiotic resistance, the stimulation of the SOS response, and *stx*-phage induction, which causes *E. coli* to release Shiga toxins and influence HUS development—although this is widely debated and controversial in the United States [269]. Freedman et al. conducted a meta-analysis in 2016 and found a significant positive association with antibiotic use and the risk of developing HUS [270], which was further strengthened by a 2018 analysis performed by Tarr et al., in which they found associations between antibiotic usage and HUS development based upon the outcome definition [271]. When ciprofloxacin was used to treat *E. coli* O104:H4 infections during the 2011 outbreak in Germany, the risk of HUS development did not increase (although only five patients were treated) [272], but O104:H4 STEC strain *stx_2_*-expression was shown to increase [273]. However, the administration of azithromycin to patients of the 2011 outbreak was associated with a reduction in O104:H4 long-term carriage [274]. The use of fosfomycin as a treatment in the 1996 Sakai, Japan O157 outbreak resulted in no apparent HUS increase in children and may have protected from HUS progression when administered within the first three days of infection [275]. Mouse model studies found that oral administration of norfloxacin, kanamycin, fosfomycin, or ampicillin early on in O157 infection alleviated infection progression [276], while gentamicin–rifampicin combination regimens above subminimum inhibitory concentrations do not induce Shiga toxin release [277]. Similar studies corroborate this notion that antibiotic subinhibitory concentrations targeting RNA polymerase, ribosomes, protein synthesis, or the cell wall, such as fosfomycin, azithromycin, gentamycin, and doxycycline, reduces *stx* production during infection [278]. Although these studies demonstrate that certain antibiotics could be used to treat STEC infections, they also made note that the *E. coli* strain, antibiotic administration timing during infection, and antibiotic concentration are important factors in treatment effectiveness [278,279].

## 7. Conclusions

STEC poses a clinical challenge in various ways through detection, isolation, and treatment. The recovery of STEC isolates in clinical settings can be challenging if there is a low bacterial concentration in patient stool samples or if the strain is of the non-O157 variety. The emergence of novel STEC strains due to acquisition of stx-phages via horizontal gene transfer and their potential impact on human health is an additional challenge, and public health can be threatened by STEC outbreaks from a combination of an infectious dose as low as 100 organisms and the lack of effective treatment regimens. The debate on antibiotics as a treatment strategy for STEC infections should be further explored to reach a definitive conclusion on their use over the course of infection. Alternative treatment strategies should be investigated to lead to a more combinatorial therapy approach. As novel and potentially infectious strains emerge, it is even more important to research effective treatment strategies to protect public health from future STEC outbreaks and infections.

## Figures and Tables

**Table 1:** Summary of bacterial AB-type toxins and representative organisms discussed in this review.

Toxin Type	Bacterium	References
**AB:**		
Botulinum toxin	*Clostridium botulinum*	[22,23]
	*Clostridium baratii*	
	*Clostridium butyricum*	
	*Clostridium argentinense*	
Diphtheria toxin	*Corynebacterium diphtheriae*	[24,25,26,27]
*Pseudomonas* exotoxin A	*Pseudomonas aeruginosa*	[28,29,30]
**AB_2_:**		
Cytolethal distending toxin (CTD)	Several Gram-negatives	[31,32,33,34,35]
	*Campylobacter jejuni*	
	*Escherichia coli*	
	*Shigella* spp.	
	*Salmonella enterica* Typhi	
	*Haemophilius ducreyi*	
	*Aggregatibacter actinomycetemcomitans*	
	*Helicobacter* spp.	
**AB_5_:**		
Cholera toxin (CT)	*Vibrio cholerae*	[36,37,38,39,40]
Pertussis toxin (Ptx)	*Bordetella pertussis*	[41,42,43,44]
ADP-ribosylating toxin (ArtAB)	*Salmonella enterica*	[45,46,47,48,49,50]
	Typhimurium	
	*Salmonella enterica* Typhi	
*Ec*Plt	Extra-intestinal *E. coli*	[51,52]
Heat-labile toxin (LT-I and LT-II)	Enterotoxigenic *E. coli*	[53,54]
EcxAB	Clinical strains of *E. coli*	[55,56]
CfxAB	*Citrobacter freundii*	[55,56]
Subtilase (SubAB)	Shiga toxin-producing *Escherichia coli* (STEC)	[57,58,59,60,61]
Shiga toxins (Stx1 and Stx2)	STEC	[62,63,64,65,66,67]
	*Shigella dysenteriae* types I and 4	
	*Shigella sonnei*	
	*Shigella flexneri*	
	*C. freundii*	

## Data Availability

Not applicable.

## Disclaimers

This research was supported by an appointment to the Agricultural Research Service (ARS) Research Participation Program administered by the Oak Ridge Institute for Science and Education (ORISE) through an interagency agreement between the U.S. Department of Energy (DOE) and the U.S. Department of Agriculture (USDA). ORISE is managed by ORAU under DOE contract number DE- SC0014664. All opinions expressed in this paper are the authors’ and do not necessarily reflect the policies and views of USDA, ARS, DOE, or ORAU/ORISE. Mention of trade names or commercial products in this article is solely for the purpose of providing specific information and does not imply recommendation or endorsement by the U.S. Department of Agriculture. USDA is an equal opportunity provider and employer.
